# Effects of Probiotic Supplementation on Exercise and the Underlying Mechanisms

**DOI:** 10.3390/foods12091787

**Published:** 2023-04-25

**Authors:** Li Zhang, Ruhao Zhang, Lu Li

**Affiliations:** 1 Department of Physical Education, China University of Mining and Technology, Beijing 100083, China; zhangli304036@126.com (L.Z.); zhangruhao992022@163.com (R.Z.); 2 School of Food and Health, Beijing Technology and Business University, Beijing 100048, China

**Keywords:** probiotics, intestinal permeability, immunity, exercise endurance, gut–brain axis, muscle metabolism

## Abstract

Long-term, high-intensity exercise can trigger stress response pathways in multiple organs, including the heart and lungs, gastrointestinal tract, skeletal muscle, and neuroendocrine system, thus affecting their material and energy metabolism, immunity, oxidative stress, and endocrine function, and reducing exercise function. As a natural, safe, and convenient nutritional supplement, probiotics have been a hot research topic in the field of biomedical health in recent years. Numerous studies have shown that probiotic supplementation improves the health of the body through the gut–brain axis and the gut–muscle axis, and probiotic supplementation may also improve the stress response and motor function of the body. This paper reviews the progress of research on the role of probiotic supplementation in material and energy metabolism, intestinal barrier function, immunity, oxidative stress, neuroendocrine function, and the health status of the body, as well as the underlying mechanisms.

## 1. Introduction

The gut microbiota plays an important role in human health and disease, and the gut microbiota status is known to be associated with dietary habits and activity levels [1]. Athletes are reported to have a higher diversity and abundance of health-promoting bacterial species in their gastrointestinal flora than sedentary individuals, associated with higher amounts of exercise and protein intake. In contrast, high carbohydrate and dietary fiber intake appear to be associated with increased abundance of *Prevotella* spp., which help break down proteins and carbohydrates [2,3]. The characteristics of the gut flora of athletes in rugby, cycling, middle-distance racing, marathon running, swimming, and rowing have been elucidated and found to show some correlation with the athletic performance of individual athletes [4]. However, prolonged strenuous exercise in athletes may also increase the stress on the gastrointestinal tract and thereby increase the probability of various symptoms associated with imbalance of the intestinal flora, such as abdominal cramps, acid reflux, emesis, and diarrhea [5,6].

**Figure 1:**
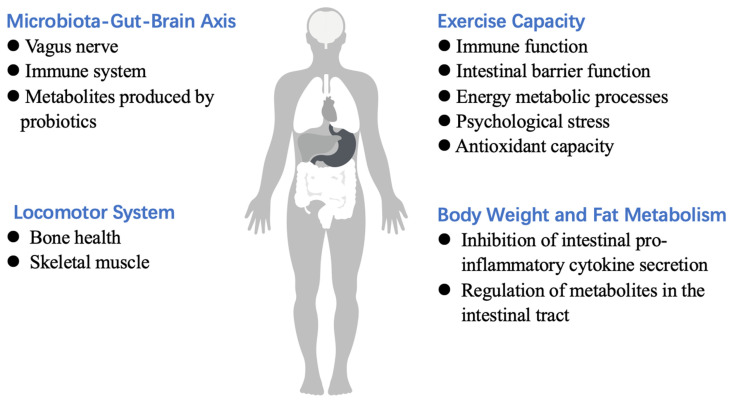
Various effects of probiotics.

Probiotics are defined as “live microorganisms that are beneficial to the health of the host at an adequate intake dose” by The Food and Agriculture Organization of the United Nations and the World Health Organization [7,8]. Probiotics have emerged as a promising treatment for alleviating gastrointestinal symptoms and improving the performance of athletes [9]. Studies have shown that probiotics have the ability to modulate the immune response, maintain the intestinal barrier, accelerate energy metabolism, prevent pathogens from adhering to host cells, ameliorate neurological diseases related to oxidative stress, and improve the production of vitamins, short-chain fatty acids (SCFAs), and neurotransmitter molecules involved in gut–brain axis communication [7,8]. In particular, the immunomodulatory effects of probiotic supplementation may help improve the defense mechanisms against upper respiratory tract infections and potentially promote the health and exercise endurance of athletes [9]. Research in the field of probiotics has made tremendous progress in the last few decades, and an increasing number of probiotic dietary supplements are available in the market [10]. The majority of probiotic strains for commercial use are from the genera *Lactobacillus*, *Bifidobacterium*, and *Bacillus* [11]. The number of products available for improving the health and athletic performance has also continued to increase dramatically. However, there is a lack of systematic review on the mechanisms underlying the effects of probiotics on exercise and how exercise performance can be improved by supplementation with probiotics. Therefore, this paper reviews the effects of probiotics on the locomotor system, locomotor ability, human body weight, fat metabolism and the underlying mechanisms; how locomotion can be influenced through modulation of the microbiota–gut–brain axis; and the methods by which probiotics can be used to improve locomotor ability and treat diseases from the perspectives of physiological metabolism, immune barrier function, and psychological stress (Figure 1). The results would be relevant and important to individuals, especially athletes, who are committed to improving their performance and health. This paper also provides a reference for subsequent research and the application of probiotics in the field of exercise.

## 2. Probiotics and the Locomotor System

### 2.1. Effect of Probiotics on Bone Health and Related Mechanisms

The bone is important for maintaining the shape of the human body and is the main attachment point of skeletal muscle. *Lactobacillus reuteri* supplementation in healthy individuals results in an increase in the level of serum 25OH vitamin D and thereby affects calcium absorption and is beneficial to bone health. Furthermore, elevated concentrations of the intestinal probiotic strains *L. reuteri* and *Bifidobacterium longum* may increase bone mineral density by promoting mineral absorption. In agreement with these results, a randomized clinical trial suggested that administration of *Lactobacillus casei* Shirota to elderly patients with distal radius fractures accelerated the healing process [12].

Probiotics play a pivotal role in bone metabolism and bone formation through immune-mediated, hormone-mediated, and nutritional mechanisms. Donkor et al. found that *Bifidobacterium* and *Streptococcus thermophilus* increased TGF-β concentrations to regulate Treg/Th17 cell differentiation, thus indirectly regulating the immune response and affecting bone metabolism [13]. The differentiation of Th17 cells is known to trigger a pro-inflammatory immune response and play a role in bone loss induced by rheumatoid arthritis and inflammatory bowel disease. Furthermore, Guss et al. found that antibiotic disruption of intestinal flora reduced CD20^+^ B and CD3^+^ T cell populations and decreased overall bone strength, thereby affecting bone remodeling and bone turnover [14]. In addition, Ohlsson et al. reported that bone mass increased in germ-free (GF) mice colonized with normal intestinal flora, mediated via NOD1 and NOD2 signaling [15].

Regarding the hormone-mediated mechanisms, it has been shown that probiotics can influence steroid hormones (e.g., estrogen and glucocorticoids), fatty acids, serotonin, and vitamin D to regulate bone remodeling. Yan et al. reported that intestinal flora stimulated bone anabolism and promoted bone formation and resorption by inducing the expression of IGF-1 [16]. In addition, Whisner et al. demonstrated that the levels of *Bacteroides* affected calcium absorption, intestinal morphology, and pH, thereby improving bone strength [17].

Overall, previous studies indicate that probiotics affect bone health through their effect via immune-related factors, such as NOD1 and NOD2 signaling, Treg/TH17 cell differentiation, and CD20^+^ B and CD3^+^ T cell populations; hormone-related factors, such as fatty acid, steroid hormones, serotonin, 25OH vitamin D, and IGF-1; and nutritional factors such as calcium absorption [18]. The effects of probiotics on bone health are shown in Table 1.

### 2.2. Effect of Probiotics on Skeletal Muscle Metabolism and Related Mechanisms

Probiotics regulate intestinal permeability through metabolites, such as SCFAs, phenolics, bile acids, and conjugated linoleic acid. These metabolites improve muscle glucose homeostasis, energy expenditure, protein synthesis, and physical activity (Table 2). Notably, these metabolites affect skeletal muscle metabolism through various pathways. Specifically, lactates produced by *Bifidobacteria* and *Lactobacilli*, can act as energy substrates for skeletal muscle [23]. Members of the phylum *Bacteroides*, such as *Prevotella copri*, produce succinate. Succinate can activate intestinal gluconeogenesis in mice fed a high-fat and high-sucrose diet, meanwhile improving glucose tolerance and insulin sensitivity in wild-type mice [24], thereby affecting skeletal muscle metabolism [25]. In addition, some *Bifidobacterium*, *Lactobacillus*, and *Bacteroides* species produce vitamin-like substances that affect energy production and storage in skeletal muscle, the interaction of skeletal muscle with the nervous system, and the interaction between muscle and bone. *Bifidobacterium* also produces conjugated linoleic acid, which induces higher expression of uncoupling protein-2 and reduces expression of fatty acid synthase and serum leptin and glucose levels. Hence, microbial-derived conjugated linoleic acid increases metabolic rates, reduces body-weight gain and white adipose tissue, thereby affecting body weight and exercise performance [26]. *Fusobacterium* can produce secondary and tertiary bile acids, which affect systemic glucose metabolism and energy expenditure, thereby influencing skeletal muscle metabolism [27].

While numerous studies have demonstrated the interaction between probiotics and skeletal muscle metabolism, the mechanisms underlying these interactions are not clear. The findings of the few mechanistic studies are presented here. Studies on GF mice have proposed that the intestinal flora affect skeletal muscle metabolism and muscle fiber type through the myenteric plexus. In GF mice exhibiting muscle atrophy, the lack of intestinal flora resulted in reduction in the expression of skeletal muscle-related genes and IGFs, and mitochondria-related functions. In contrast, transplantation of feces from pathogen-free mice led to an increase in skeletal muscle mass, a decrease in markers of skeletal muscle atrophy, and improvement in the oxidative metabolism of muscle.

In addition, Jollet et al. reported that severe physical inactivity for a short period led to muscle atrophy and exerted adverse effects on *Clostridium* populations [28]. Specifically, *Spirllaceae* species can produce SCFAs and convert primary bile acids to secondary bile acids. Therefore, *Spirllaceae* species may be generally sensitive to low activity levels and may play a key role in the effect of low activity on intestinal flora. The probiotics, their metabolites, and their function related to skeletal muscle metabolism are summarized in Table 2.

**Table 2:** Effects of probiotic metabolites on skeletal muscle metabolism.

References	Probiotic	Metabolite of Probiotic	Effect
[29]	*Lactobacillus, Bifidobacterium*	Lactic acid	Energy substrate
[30]	Most bacteria, fibrinolytic bacteria, glycolytic bacteria, protein hydrolytic bacteria	Short-chain fatty acids	Systemic insulin resistance, inflammation, appetite, muscle insulin sensitivity, muscle atrophy, muscle strength, and exercise capacity
[31]	Butyrate-producing probiotics	Phenolic metabolites	Glucose uptake and metabolism in human skeletal muscle myoblasts
[26]	*Bifidobacterium*	Conjugated linoleic acid	Weight and physical performance
[32]	*Clostridium*	Secondary and tertiary bile acids	Systemic glucose homeostasis and energy consumption
[33]	*Bifidobacterium, Lactobacillus*	B group vitamins and short chain fatty acids	Energy metabolism and host energy intake
[34]	*Propionibacterium shermani*	Vitamin B_12_	Improvement of energy and exercise tolerance, and alleviation of fatigue and shortness of breath
[35]	*Bacteroides*	Vitamin K_2_	Improvement of bone mineral density

## 3. Interrelationship between Probiotics and Exercise Capacity

### 3.1. Effect of Probiotics on Exercise Capacity

Probiotics can affect exercise capacity by reducing stress injury and exercise fatigue, as well as increasing the duration of exhaustive exercise and endurance time. Hsu et al. found that both specific pathogen-free (SPF) and *Bacteroides fragilis* (BF) mice had longer endurance swimming times than GF mice [36]. The inoculation of atypical *Veillonella* from long-distance runners into mice also significantly prolonged the duration of exhaustive exercise, indicating this specific strain has the potential to improve locomotor performance. Thus, probiotics have potential applications in increasing the duration of exhaustive exercise and endurance time, as well as improving athletic performance. However, current research results are mainly based on animal experiments, and the long-term intervention effects of probiotics and their mechanisms in athletes are still unclear. Therefore, there is an urgent need to conduct large-scale, long-term randomized controlled studies to clarify the intervention effects and provide evidence for the application of probiotics.

### 3.2. Mechanisms by Which Probiotics Affect Exercise Capacity

Probiotics can enhance exercise capacity by improving host immune function, intestinal barrier function, energy metabolic process, psychological stress, and antioxidant capacity (Table 3). These effects may be related to the ability of probiotics to metabolize carbohydrates to produce SCFAs, including acetic acid, butyric acid, and propionic acid. These effects are described in detail below.

#### 3.2.1. Regulation of Metabolism

Probiotics can regulate glucose metabolism, lipid metabolism, protein and amino acid metabolism, as well as vitamin metabolism to compensate for the energy deficit generated by intense exercise. Hence, probiotics play an important role in maintaining the energy supply balance for the body. For example, changes in bile acids caused by intestinal microorganisms can improve glucose tolerance and control glucose homeostasis [45]. Nay et al. studied the effects of 21 days of mixed treatment with broad-spectrum antibiotics and 10 days of natural replanting and found that markers of glucose metabolism, such as SCFAs, G protein-coupled receptor 41, sodium-glucose co-transport protein 1, and myogenic genes, in the ileum were associated with the changes in muscle endurance observed after the treatment [46]. Furthermore, probiotic metabolite isovanillic acid 3-O-sulfate increased glucose transport factor 1, glucose transport factor 4, and phosphatidylinositol-3-hydroxyl kinase activity, while promoting phosphorylation of threonine kinase [47]. These findings imply that the intestinal flora can regulate glucose metabolism in myoblasts. Thus, changes in the intestinal flora may be closely related to glucose metabolic process that is important for restoring muscle endurance, regulating muscle metabolism, and thereby affecting energy production in muscle.

SCFAs produced by gut microbial degradation are involved in host energy metabolism, and improve myocyte energy production efficiency by regulating mitochondrial biosynthesis [48], meanwhile increase protein expression of PGC-1α and uncoupling protein 1 in brown adipose tissue, thereby accelerating thermogenesis and fatty acid oxidation [49]. Mardinoglu et al. compared the metabolic differences between GF and conventional mice and found differences in the content of free amino acids in their gastrointestinal tract [50]. Accordingly, changes in bile acids caused by intestinal metabolic microorganisms have been found to promote fatty acid uptake [51]. In accordance with these findings, studies on supplementation with probiotics have shown that they can increase the concentration of free amino acids, bioactive peptides, γ-aminobutyric acid, and other nutrients and metabolites through enzymatic breakdown of proteins, as well as play a role in host energy metabolism [52,53]. Furthermore, probiotic BC30 administration improved protein absorption and increased vertical jumping ability [40]. Similarly, *Lactobacillus plantarum* PS128 supplementation in triathletes significantly increased the levels of plasma branched chain amino acids [41]. These studies suggest that probiotics can mediate in vivo protein and amino acid metabolism to affect exercise capacity.

Li et al. conducted whole-genome analysis and found that *Lactobacillus plantarum* ZJ316 contains 23 protease genes that encode for most amino acids, except for valine, leucine, and isoleucine [39]. Their findings imply that probiotic bacteria affect the protein hydrolase system and amino acid biosynthesis. In addition, a human study involving good rugby players found a positive correlation between gut microbial diversity index and protein intake, as well as creatine kinase concentration [54]. Probiotics can also regulate vitamin B metabolism and thus affect energy metabolism under in vivo conditions. Vitamin B, as a cofactor, may be directly involved in the energy production function of the respiratory chain. In accordance with these findings, Cárdenas et al. isolated *Lactobacillus fermentum* CECT5716 from healthy breast milk and sequenced the whole genome, and they found that the strain contained gene clusters for riboflavin and folate biosynthesis [55]. Furthermore, Magnúsdóttir et al. analyzed the genome of 256 strains from human intestinal bacteria using the PubSEED platform and predicted that 40% to 65% of people have intestinal flora with biosynthetic pathways for eight B vitamins [43]. Effects of probiotics on regulation of metabolism are shown in Table 4.

#### 3.2.2. Enhancement of Immune Function

Vigorous exercise or overload training predisposes individuals to upper respiratory tract infection (URTI) and gastrointestinal discomfort [58,59]. However, probiotics may modulate the immune system to reduce the incidence, duration, and severity of URTIs, and this may have an indirect effect on improving training or competition performance [60]. This has been demonstrated in studies by Cox et al. and West et al., who supplemented *L. fermentum* VRI-003 (PCC) to long-distance runners and cyclists, respectively, and Gleeson et al., who supplemented *L. casei* Shirota to endurance athletes. All three studies showed that probiotics could reduce the incidence and degree of URTIs by improving mucosal immune function in athletes [61,62,63]. In addition, multiprobiotic supplementation over a 3-month period of winter training significantly reduced the incidence of URTI in athletes after fatiguing aerobic exercise [64]. 

The ameliorative effect of probiotics on respiratory diseases is mainly achieved through stimulation/modulation of the immune system (Table 5). Probiotics enhance innate immunity by upregulating phagocytic and natural killer cell activity and enhancing acquired immunity by improving antigen presentation and T- and B-lymphocyte function [65]. In addition, probiotic supplementation may also have an immunomodulatory effect by reducing the secretion of inflammatory factors and inducing an anti-inflammatory response. Specifically, supplementation with *Lactobacillus casei* Shirota daily for 30 days prior to a marathon improved systemic and airway immune responses reduced levels of pro-inflammatory cytokines and elevated levels of anti-inflammatory cytokines in the upper airways in male marathon runners [66].

Probiotics can enhance intestinal mucosal immune function by regulating intestinal flora, enhancing intestinal mucosal barrier function, and inhibiting the expression of inflammatory factors. Intense exercise or overtraining can cause inadequate perfusion of the digestive tract, disrupting the integrity of the mucosal barrier and leading to increased intestinal permeability, which, in turn, induces an inflammatory response and increases the risk of intestinal-related diseases [75]. Probiotic supplementation has been shown to be an effective and safe way to prevent and treat exercise-induced gastrointestinal symptoms [76]. Specific genera, such as *Lactobacillus* and *Bifidobacterium*, as well as specific species such as *Lactobacillus rhamnosus*, can improve the gastrointestinal discomfort caused by a single bout of high-intensity or endurance exercise. It has also been shown that probiotic supplementation can reduce exercise-induced increases in intestinal permeability and maintain intestinal mucosal barrier integrity. For example, Lamprecht et al. found that supplementation with multi-species probiotics for 14 weeks reduced fecal levels of zonulin (a marker of intestinal mucosal barrier function) in male athletes after a single bout of high-intensity exercise [77]. Further, 11 weeks of *Lactobacillus fermentum* supplementation in bicyclists was found to result in a 7.7-fold increase in the *Lactobacillus fermentum* population in the stool, and the extent of probiotic colonization in the intestine was directly proportional to the reduction of gastrointestinal symptoms [62]. In addition, probiotics may also improve intestinal defense by upregulating the expression of tight junction proteins and promoting mucus synthesis [78,79].

Inhibition of inflammatory factor expression and promotion of SIgA secretion are important ways for probiotics to improve intestinal immune function. Probiotics regulate the secretion of cytokines, such as NF-κB, MAPK, and PKC, as well as signal transducers and activators of transcription pathways in intestinal epithelial cells, macrophages, and dendritic cells by modulating key signaling pathways [80]. Specifically, probiotics, such as *L. acidophilus* and *Bifidobacterium bifidum*, were found to alleviate intestinal mucosal inflammatory damage by reducing the expression of inflammatory factors TNF-α, IL-6, IL-1β, IL-8, and neutrophil infiltration, while also decreasing intestinal permeability [81]. In addition, probiotics may improve the function of the intestinal immune barrier by promoting IgA secretion from intestinal plasma cells, thus preventing the proliferation of pathogens in the intestine. This is demonstrated in the study of Kabeerdoss et al., who supplemented young healthy women with yogurt containing *Bifidobacterium lactis* Bb12 for 3 weeks and found a significant increase in fecal IgA levels during the period of probiotic supplementation [82].

#### 3.2.3. Improvement of Oxidative Stress

High-intensity exercise generates a variety of free radicals and reactive oxygen species, which cause oxidation of proteins, lipids, and nucleic acids, promote apoptosis, and impair cellular function. The degree of oxidation has been found to be negatively correlated with intestinal *Lactobacillus* and *Bifidobacterium* populations [83]. Accordingly, Hsu et al. found that SPF mice had higher serum glutathione peroxidase and catalase levels than GF mice; furthermore, serum glutathione peroxidase levels were higher in BF mice than in GF mice [36]. In addition, *Lactobacillus paracasei* and *Lactobacillus rhamnosus* administered to athletes during 4 weeks of vigorous exercise exerted strong antioxidant activity and resulted in an increase in plasma antioxidant levels [84]. Thus, it appears that gut microbial deficiency results in a reduction in the activity of antioxidant enzymes, while probiotics can regulate intestinal flora homeostasis and reduce the level of oxidative stress [85].

The mechanisms underlying the effect of probiotics against oxidative stress include chelation of metal ions, maintenance of antioxidant enzyme systems, metabolization and production of antioxidant substances, and mediation of antioxidant metabolic pathways [86,87,88,89]. Overall, the antioxidant effects of probiotics can slow down the oxidative damage caused by free radicals generated during exercise, reduce apoptosis, and enhance exercise capacity.

#### 3.2.4. Improvement of Intestinal Barrier Function

Intestinal flora homeostasis is one of the main factors affecting intestinal barrier function, and intestinal flora and metabolites are important mediators that affect intestinal mucosal barrier integrity and intestinal permeability. High-intensity exercise causes stress in the gastrointestinal tract and may lead to endotoxemia in severe cases, but probiotics can prevent ischemia-induced gastrointestinal problems by improving the mucosal and epithelial barriers to prevent “leaky gut” and by producing anti-inflammatory mediators [90]. Probiotic supplements containing *L. rhamnosus*, *L. fermentum*, or multiple strains resulted in slight to moderate improvements in the severity and duration of gastrointestinal problems [91,92]. Studies on the effects of probiotics on intestinal barrier function in athletes have shown positive intervention effects [93]. For example, probiotic supplementation reduced the amount of zonulin in the stool of endurance athletes, and zonulin is considered an indicator of increased intestinal permeability [68]. In addition, probiotics were found to reduce plasma d-lactate levels and improve intestinal mucosal barrier function in patients with inflammatory bowel disease. Furthermore, Roberts et al. found that probiotic supplementation 12 weeks before a triathlon reduced gastrointestinal symptoms and decreased endotoxin levels in the body [94]. Meanwhile, probiotics also have direct antibacterial activity and regulatory effects, which can alleviate the gastrointestinal barrier damage caused by high-intensity exercise by promoting the synthesis of intestinal mucosal glycoproteins and enhancing the protective effect on the mucosal layer.

#### 3.2.5. Alleviation of Psychological Stress

Recent studies have shown that the intestinal flora are closely related to the development of the neuroendocrine system. However, there are few studies on the improvement of psychological stress by probiotic supplementation. Nonetheless, the available evidence from animal and human studies suggests that probiotics may have beneficial effects on psychological well-being in humans [95]. For example, Li et al. found that probiotics attenuated depressive behavior in rats by remodeling intestinal flora, increasing norepinephrine and 5-hydroxytryptamine levels, and inhibiting adrenocorticotropic hormone and corticosterone expression [96]. Additionally, Michalickova et al. evaluated excellent athletes supplemented with *Lactobacillus helveticus* for 14 weeks before and after treatment by self-assessment of their emotional state, and their results showed that the probiotic supplementation group had an increased sense of self-assessed vitality compared to the placebo group [97].

Although it has been shown that probiotic supplementation significantly reduces preclinical psychological symptoms, such as anxiety, depression, and stress, in healthy individuals, this effect may be reduced in individuals with chronic diseases (as a result of alteration in intestinal flora and immune function and the presence of psychological co-morbidities) [98]. Currently, most of the research on probiotics in neurology is focused on psychological conditions such as depression and anxiety disorders [99].

## 4. Mechanisms by Which Probiotics Improve Body Weight and Fat Metabolism

Obesity is often accompanied by a series of metabolic syndromes, such as hypertension and atherosclerosis. In recent years, numerous studies have shown that intestinal microorganisms and low levels of inflammation in the intestine are important causes of obesity and related diseases [100]. Probiotics can regulate the composition of intestinal flora, inhibit the growth of harmful bacteria and the production of harmful metabolic substances and pro-inflammatory cytokines, promote carbohydrate metabolism, and regulate immune process [101,102,103]. Hence, probiotics may prevent and control obesity caused by dysbiosis of intestinal flora and long-term inflammation in the intestine.

### 4.1. Inhibition of Intestinal Pro-Inflammatory Cytokine Secretion

Obesity is a chronic inflammatory condition, and the gut is the site of inflammatory cytokine production. When inflammatory symptoms appear in the intestine, the content of pro-inflammatory cytokines in the small intestine is significantly increased. The levels of IL-6, IL-8, IFN-γ, MCP-1, and CRP were found to be higher in obese children than those with normal BMI [104]. This finding supports the notion that obesity leads to an increase in the secretion of pro-inflammatory factors. Thus, probiotics may play a role in reducing body weight by inhibiting the secretion of intestinal pro-inflammatory factors and decreasing intestinal inflammation (Table 6).

### 4.2. Regulation of Metabolites in the Intestinal Tract

#### 4.2.1. Effect of SCFAs

Carbohydrates are hydrolyzed in an anaerobic environment in the colon to generate monosaccharides, which are then used to generate SCFAs using phosphoenolpyruvate, an intermediate product within the glycolytic pathway process. SCFAs can act as ligands to activate the G protein-coupled receptors GPR41 and GPR43. GPR41 stimulates the secretion of glucagon-like peptide from enteroendocrine L cells, and GPR43, activated at the cellular level, increases the secretion of GLP-1. Both GPR41 and GPR43 regulate blood glucose levels and improve lipid metabolism, while suppressing appetite, producing a feeling of satiety, and reducing body weight [108]. In addition, butyrate produced from SCFAs not only has anti-inflammatory effects but also increases the production of IgA [109]. Thus, the intake of probiotics may have lipid-lowering effects via the stimulation of GLP-1 release and the upregulation of butyrate concentrations (Table 7).

#### 4.2.2. Effect of Bile Acids

Bile acids promote small intestinal lipid metabolism and act as ligands to activate farnesol X receptor (FXR) and G protein-coupled receptor (TGR 5), which regulate lipid metabolism and energy conversion. When FXR levels are low, triglyceride and cholesterol levels in the liver increase and induce atherosclerosis. TGR 5 activates adenylate cyclase to convert ATP to cAMP, which induces protein kinase A to activate the cAMP response element-binding protein, thereby stimulating various cAMP signaling pathways [110]. The consumption of probiotics can alter the bile acid metabolic process and thus affect lipid metabolism and body weight (Table 7).

**Table 7:** Effects of probiotics and metabolites.

Intestinal metabolites		**References**	**Probiotic**	**Study Design**	**Conclusion**
	SCFAs	[111]	*Lactobacillus plantarum* ZJUFT17	Influence of GML and the *Lactobacillus plantarum* on body weight, serum lipid profiles, inflammatory responses and gut microbiota	*Lactobacillus plantarum* ZJUFT17 inhibited weight gain, decreased total cholesterol, decreased serum TNF-α level, and increased α-diversity of intestinal flora in mice
		[112]	*Lactobacillus rhamnosus* LS-8, *Companilactobacillus crustorum* MN047	Intervention with *Lactobacillus rhamnosus* and *Companilactobacillus crustorum* in mice on a high-fat diet	Increase in the concentration of butyric acid and decrease in the body weight of mice
	BAs	[113]	Lab4 probiotics and *Lactobacillus plantarum* CUL66	Cholesterol-lowering effects of short-term feeding of Lab4 probiotics and *Lactobacillus plantarum* CUL66 in wild-type mice	Probiotics hydrolyze bile salts, assimilate cholesterol, and regulate cholesterol transport by polarizing Caco-2 intestinal cells
		[114]	*Lactobacillus plantarum* K21	In vitro screening of 88 strains of probiotics	Hydrolyzation of bile salts; decrease in cholesterol levels; inhibition of lipid accumulation in 3T3-L1 preadipocytes; increase in intestinal permeability
		[115]	*Lactobacillus*	Investigation of the improvement of probiotics on HFD and HSD induced microflora disorders	Probiotics may alleviate diet-related obesity by regulating gut flora

SCFAs = short-chain fatty acids; BAs = bile acids; HFD = high-fat diet; HSD = high-sucrose diet.

## 5. Role of Probiotics in Movement via the Microbiota–Gut–Brain Axis

The continuous research on intestinal flora over the last few decades has revealed that the gut and brain can be regulated in both directions through the gut–brain axis (GBA). Further, research on the effects of probiotics on exercise performance and the GBA has led to the new concept of the “microbiota–gut–brain” (MGB) axis [116]. The pathways identified in the MGB axis include the vagus nerve, the immune system, and the metabolites produced by the intestinal flora [117]. Although studies have been conducted on the ways in which the MGB axis regulates specific movements, most of them do not provide a detailed picture of the mechanisms involved, and the more intrinsic connections need to be further investigated.

Probiotics have an important impact on human cognitive function. Studies have shown that the intestinal flora can influence the brain function of the host through the GBA and promote brain plasticity. For example, Savignac et al. showed that *B. longum 1714* administration exerted a positive impact on cognition in an anxious mouse strain [118]. Additionally, Cotman et al. found that exercise also improves learning memory function by regulating the levels of learning memory-related molecules such as N-methyl-d-aspartate receptors and brain-derived neurotrophic factors in the brain [119]. Although more studies in this area are needed, the evidence available indicates that exercise may affect brain behavior through the GBA.

## 6. Probiotic Treatment Methods for Improving Exercise Capacity and Treating Disease

The prominent probiotic treatment methods for regulating intestinal flora, improving mobility, and curing or managing diseases include changes in dietary structure, intake of probiotics and prebiotics, fecal transplantation, and traditional techniques from Chinese medicine (Table 8).

### 6.1. Dietary Structure

Changes in dietary structure can affect the composition and function of the intestinal flora, and the metabolites synthesized by the intestinal flora have an impact on the physiological and biochemical responses of the host and are important for establishing an interactive and interdependent system between the host and the intestinal flora [139]. Abundance of *Erwinia* is positively correlated with salt intake, and high salt intake leads to an increase in cellular pro-inflammatory factors, which, in turn, produce an inflammatory response that disrupts the intestinal environment and increases blood pressure [140]. In addition, a high salt diet may also elevate blood pressure by affecting the metabolic pathways of specific flora and activating salt corticosteroids [141]. On the other hand, a diet that is high in dietary fiber has been found to increase the abundance of acetate intestinal bacteria in hypertensive mice and reduce pathological features, such as cardiac hypertrophy and fibrosis [142]. Furthermore, comparison of the intestinal flora of vegetarians with that of individuals who consume meat showed that spore-forming fungi and edible bacteria (*Fusarium* and *Penicillium*) were more abundant in the intestinal flora of vegetarians [143]. In addition, a high-fat diet resulted in a significant increase in the abundance of *Lactobacillus* and a drastic decrease in that of *Clostridium* in the small intestine of mice, as well as pathophysiological alteration of the luminal environment in the small intestine [144]. As probiotics are known to affect exercise capacity, these findings lay the basis for future studies on the relationship between dietary structure, exercise capacity, and the role of the MGB axis.

### 6.2. Intake of Probiotics and Prebiotics

The probiotics currently approved for humans are *Bifidobacterium*, *Lactobacillus*, *Enterococcus*, *Escherichia coli*, and *Bacillus subtilis*, among others. Prebiotics are indigestible food ingredients, mostly composed of carbohydrates, and are mostly found in natural products such as fruits, vegetables, and grains [145]. Some common prebiotics include inulin, mucin, lactulose, goat milk oligosaccharides, polydextrose, gum arabic, and guar gum. Undigested prebiotics are transported to the large intestine, where they are degraded and utilized by the intestinal flora. The secondary metabolites produced are absorbed by the intestinal epithelium or transported to the liver via the portal vein, ultimately affecting the physiological processes of the body [146].

The intake of probiotics and prebiotics reduced the incidence and symptoms of URTIs, decreased gastrointestinal symptoms and intestinal mucosal barrier permeability, enhanced physical capacity, promoted post-exercise recovery, and improved mental stress in athletes [147]. However, the mechanism via which probiotic supplementation improves athletic performance in athletes is still controversial and needs to be investigated further [148]. In addition, although numerous studies have confirmed the benefits of specific microbial species, there are still several partially characterized probiotic strains that need to be investigated for their effects on the human body [149,150]. The benefits of probiotics may not always be evident under conditions of intense exercise, which can cause abnormalities in body function and the intestinal environment. Furthermore, immune deficiencies caused by intense exercise may lead to some probiotic strains becoming opportunistic pathogens and leading to life-threatening diseases [151]. In addition, it has been shown that probiotic intake can inhibit the accumulation of tubular and mucosal microbiota in human and mouse intestines [152]. Thus, probiotics species that show potential need to be carefully investigated before they are approved for use in supplements.

### 6.3. Fecal Microbiota Transplantation

Fecal microbiota transplantation (FMT) refers to the transplantation of functional flora from the feces of healthy donors into the gastrointestinal tract of patients to reconstitute the intestinal flora of patients and make it function normally for the treatment of intestinal and extraintestinal diseases [153]. FMT has been found to be effective in the treatment of *Clostridium difficile* infection and has been used in numerous clinical trials on the treatment of other diseases. Smits et al. evaluated vascular inflammatory markers in 20 men with obesity who had cardiometabolic syndrome and found that FMT with lean donors temporarily altered the intestinal flora in the patients with obesity [154]. At present, most FMT studies are in the form of clinical case reports or case series, and the findings indicate that the clinical efficacy varies by individual and disease. Bacterial transplantation alone cannot fully explain the differences in treatment outcomes [155]. Furthermore, a study on FMT for the treatment of patients with recurrent *C. difficile* infection demonstrated overrepresentation of certain fungi in FMT recipients or donors may reduce the therapeutic efficacy [156].

The current FMT technology is still in its early stages, and the effects of different donor intestinal flora on the treatment of different diseases need to be studied in more depth. Moreover, efforts to standardize FMT technology should be continued to achieve standardization of donor selection, sample handling, and immunocompatibility between donors and recipients. Studies on FMT safety are also important, as a study reported that two patients with *Clostridioides difficile* infection developed bacteremia after transplantation of *E. coli* and one of them died [157].

### 6.4. Chinese Medicine

Certain Chinese medicines help prevent obesity and various diseases by improving the structure of intestinal flora, increasing the amount of probiotics, and reducing pathogenic bacteria populations to inhibit weight gain and alleviate endotoxemia and insulin resistance [158]. Aqueous extract of *Ganoderma lucidum* induced weight loss, improved inflammation, increased insulin sensitivity, and reversed ecological disorders of the intestinal flora in mice with obesity [123]. Polysaccharides from *Ophiopogon japonicas* were found to decrease the *E. coli* and *Streptococcus* populations and increase the *Bifidobacterium* populations in the intestinal flora of mice with diabetes [159]. In addition, *Portulaca oleracea* was found to promote the growth of *Bifidobacteria*, reduce excessive immune response in the intestine, and regulate intestinal microecological disorders [160].

Another traditional technique in Chinese medicine is acupuncture, which is a general term used to refer to acupuncture manipulation and moxibustion. It has been shown that acupuncture can improve the number and type of intestinal flora, and this can play a role in the treatment of type II diabetes [161]. Different meridian points and acupuncture techniques can affect the intestinal flora in different ways, and this can improve gastrointestinal, metabolic, and immune diseases in humans [162].

## 7. Conclusions and Future Prospects

Probiotics play a pivotal role in the maintenance of health through their effects on the regulation of bone and skeletal muscle, the metabolism of energy and various nutrients, the inflammatory response, the intestinal barrier function, the immune function, the improvement of oxidative stress, and the alleviation of psychological stress, thereby reducing exercise fatigue and improving exercise performance. Therefore, probiotic supplements have significant market value in various groups of individuals—from elite athletes to patients with obesity and obesity-related conditions. However, the research in this area needs further development in terms of the identification of beneficial strains with a safety profile and detailed dose response, underlying mechanisms, standardization of treatment protocols, and best practices to advance the application of probiotics as functional products.

## Figures and Tables

**Table 1:** Effects of probiotics on bone health.

References	Probiotic	Study Design	Conclusion
[12]	*Lactobacillus casei* Shirota	Elderly patients with an acute distal radius fracture supplemented with 6 × 10^9^ CFU daily for 6 months	DASH score, pain, CRPS score, wrist flexion and grip strength exhibited a significantly faster pace of improvement
[19]	*Bifidobacterium longum*	Supplementation to male Wistar rats for 4 weeks	Increase in tibia Ca, P, Mg content and fracture strength
[20]	*Lactobacillus reuteri*	Supplementation to adult male mice 3 times a week for 4 weeks	Increase in femoral and vertebral bone formation and trabecular bone volume
[21]	*Lactobacillus reuteri*	Older women supplemented with 10^10^ CFU daily for 12 months	Reduction in the loss of total volumetric bone mineral density
[22]	*Lactobacillus reuteri*	Older women supplemented with 10^10^ CFU daily for 12 months	Prevention of a deterioration of the gut microbiota and inflammatory status and beneficial effects on bone metabolism

DASH = disabilities of the arm, shoulder, and hand; CRPS = complex regional pain syndrome.

**Table 3:** Effects of probiotics on exercise capacity.

References	Probiotic	Study Design	Conclusion
[36]	Intestinal flora	Broad-spectrum antibiotic intervention in mice for 21 days followed by a 10-day period for natural recovery of intestinal flora	Glucose metabolism markers, such as GPR41 and SGLT-1 gene expression and changes in myoglycogen levels, indicate effective restoration of muscle endurance
[37]	*Lactobacillus plantarum* TWK10	2.05 × 10^8^ CFU/kg administered to 6-week-old male ICR mice	Decrease in blood lactate and blood glucose concentrations after exercise; improvement in glucose utilization; increase in the number of gastrocnemius type I muscle fibers and lean body mass in rats; decrease in blood ammonia and creatine kinase levels; resistance to fatigue; improvement in exercise capacity
[38]	Intestinal flora	Sodium taurocholate (3 mg/g of body weight) and lithocholic acid (0.1 mg/g of body weight) for 5 days	Stimulation of the secretion of fibroblast growth factor 15; inhibition of AgRP/NPY neurons; improvement in glucose tolerance and control of glucose homeostasis
[39]	*Lactobacillus plantarum* ZJ316	Comparative genomic analysis	34 genes encoding intracellular peptidases and 23 genes encoding proteases of different specificity
[40]	Probiotics BC30	20 g of casein plus probiotics twice daily and full body exercise 4 times a week for 8 weeks in a group of 10 healthy resistance-trained individuals	Improved exercise performance, specifically, an increase in the vertical jumping ability of subjects
[41]	*Lactobacillus plantarum* PS128	Capsules containing 300 mg of lyophilized bacterial powder, equivalent to 1.5 × 10^10^ CFU, taken by 18 subjects recruited from the triathlon team	Significant increase in the level of plasma branched-chain amino acids by 24–69%, significant increase in exercise performance, and effective improvement in the dynamic balance of muscle energy metabolism during exercise
[42]	*Lactobacillus casei*	20 young male soccer players randomly assigned to consume a commercially available probiotic drink (Yakult) containing 6.5 × 10^9^ CFU per bottle for one month, twice daily	Significant increase in the phenylacetylglutamine level after correction for creatinine levels, and decrease in the ammonia level
[43]	Intestinal flora	Genome analysis of 256 strains of human intestinal bacteria through the PubSEED platform	Intestinal flora with biosynthetic pathways for the synthesis of eight B vitamins detected in 40% to 65% of individuals
[44]	*Lactobacillus plantarum* CRL 725	Quantitative analysis of riboflavin	Significant increase in the concentration of riboflavin

**Table 4:** Effects of probiotics on regulation of metabolism.

References	Probiotic	Regulation of Metabolism
[41]	*Lactobacillus plantarum*	Increase of plasma-branched amino acids and improvement of exercise performance
[48]	Probiotics that produce short-chain fatty acids	Source of host energy and improvement of insulin sensitivity and energy expenditure
[50]	Probiotics that modulate host amino acids	Modifications in glutathione metabolism
[52]	*Lactiplantibacillus plantarum*	Enhancement of digestion and absorption of nutrients, and stimulation of the synthesis of beneficial compounds
[56]	*Bifidobacterium* and *Lactobacillus*	Improvement in glucose metabolism in pregnant women with diet-controlled gestational diabetes mellitus
[57]	*Lactobacillus plantarum*	Improvement in gut microbiota structure and lipid metabolism in mice fed high fat diet

**Table 5:** Mechanisms by which probiotics enhance immune function.

References	Probiotic	Study Design	Conclusion
[67]	*Lactobacillus helveticus* Lafti L10	Supplement for outstanding athletes during winter training	Increase in the CD4^+^-to-CD8^+^ ratio; reduction in the duration of respiratory infection symptoms; no effect on the severity and morbidity of respiratory disease
[68]	Several *Lactobacillus* species	Four-week probiotic supplementation in elite rugby league players	Decrease in the incidence of URTIs and the number of days of illness; significant decrease in the incidence of gastrointestinal diseases; no effect on the severity of respiratory symptoms
[69]	*Lactococcus*	14-day course for male college athletes	Reduction in the incidence of URTIs and relief in symptoms, such as sneezing and runny nose
[54]	Multiple probiotics (*Bifidobacterium, Bifidobacterium lactis*, *Enterococcus faecium*, *Lactobacillus acidophilus*, *Lactobacillus brevis*, *Lactococcus lactis*)	Supplementation during 3 months of winter training for athletes	Significant reduction in the incidence of URTIs after fatiguing aerobic exercise
[70]	*Lactobacillus casei* Shirota	Daily supplement taken for 30 days prior to a marathon by male marathoners	Decrease in nasal mucosal neutrophil infiltration; decrease in the levels of pro-inflammatory cytokines (such as IL-1, IL-5, IL-6, IL-13, and TNF-α) in the upper respiratory tract; increase in the levels of anti-inflammatory cytokines (IL-10); maintenance of salivary IgA levels after a marathon
[71]	*Bifidobacterium animalis*, *Lactobacillus acidophilus*	Male marathoners randomly received *Bifidobacterium*, *Lactobacillus acidophilus* or placebo treatment daily for 30 days prior to a race	Decrease in the production of pro-inflammatory cytokines by lymphocytes after the marathon; maintenance of the number of CD8 T cells and effector memory cells; and immunomodulatory effect via the stimulation of lymphocytes
[72]	*Lactobacillus acidophilus*	Capsules containing 2 × 10^10^ CFU of *Lactobacillus acidophilus* taken daily for 30 days by athletes	Increase in IFN-γ production by T cells in fatigued athletes and increase in saliva IFN-γ concentrations in non-fatigued athletes The ability of *Lactobacillus acidophilus* to reverse T-cell defects and enhance mucosal IFN-γ concentration was demonstrated, and its effects may be related to the immune status of the organism
[51]	*Lactobacillus fermentans* VRI-003 PCC^®^	1.26 × 10^10^ CFU as lyophilized powder in gelatin capsules taken daily over 4 months of winter training by 20 healthy excellent male distance runners	Decrease in the duration of respiratory symptoms (30 days) to less than half; decrease in the disease severity of episodes during treatment; two-fold increase in whole-blood culture IFN-γ levels
[52]	*Lactobacillus fermentans* VRI-003 PCC^®^	One probiotic capsule containing at least 1 × 10^9^ CFU of *Lactobacillus fermentum* VRI-003 PCC^®^ taken daily by competitive bicyclists	Significant reduction in the severity of self-reported symptoms and the disease load of lower respiratory disease in male athletes; significant reduction in the severity of gastrointestinal symptoms at higher training intensities
[54]	*Lactobacillus casei* Shirota	Two cans of a probiotic drink containing at least 6.5 × 10^9^ LcS live cells consumed daily for 16 weeks by 20 healthy excellent male distance runners	Significant decrease in the proportion of subjects presenting with URTI symptoms for 1 week or more; significant reduction in the number of URTI episodes; increase in salivary IgA concentration and significant treatment effect
[73]	*Bacillus subtilis* DE111	25 male athletes randomized to a probiotic and placebo group for a 12-week intervention study	Significant decrease in TNF-α concentration in the probiotic group compared to the placebo group
[74]	*Lactobacillus salivarius* UCC118	UCC118 or placebo supplementation daily for 4 weeks in 7 healthy adults	Significant reduction in the abundance of *Verrucomicrobia* and a significant increase in the abundance of butyric acid-producing Rosehips and *Lachnospiraceae bacterium*; amelioration of exercise-induced increase in intestinal permeability and remodeling of the intestinal microbiome

**Table 6:** Effects of probiotics on intestinal pro-inflammatory factors.

References	Probiotic	Study Design	Conclusion
[44]	*Lactobacillus plantarum*, *Lactobacillus fermentans*	Intervention with *Lactobacillus plantarum* and *Lactobacillus fermentum* alone or in combination in rats on a high-fat diet	*Lactobacillus plantarum*-induced reduction in the levels of the pro-inflammatory cytokine IL-6 and endotoxins
[105]	*Lactobacillus fermentans* CQPC07	Intervention with *Lactobacillus fermentum* CQPC07 in rats on a high-fat diet	Decrease in the levels of pro-inflammatory cytokines (IL-6, IL-1β, and TNF-α); increase in the levels of anti-inflammatory cytokines (IL-4, IL-10)
[106]	*Lactobacillus curvatus* HY760, *Lactobacillus plantarum* KY1032	Simultaneous intervention with two probiotic strains in mice with fat diet-induced obesity	Reduction in the body weight of mice; decrease in the content of pro-inflammatory cytokines (IL-6, IL-8, and IL-1β)
[107]	*Lactobacillus* OK67	*Lactobacillus* OK67 gavage in mice on a high-fat diet	Inhibition of the expression of pro-inflammatory factors (IL-1β, TNF-α, and NF-KB) in the colon; increase in the expression of anti-inflammatory factors (IL-10) and tight junction proteins

**Table 8:** Effects of probiotics on improving exercise performance and ameliorating disease.

References	Supplementation /Methods	Study Model	Related Diseases/ Manifestations	Conclusion
[120,121,122]	Berberine	Mice	Obesity	Improvement in the *Bacteriodetes*-to-*Firmicutes* ratio; increase in the *Lactobacillus* and *Allobaculum* population; decrease in the *Lachnospira* and *Clostridium* population in mice with HFD-induced obesity
[123]	*Ganoderma lucidum*	Mice	Obesity, IR, T2DM	Reduction in body weight; reduction in inflammation; increase in insulin sensitivity; reversal of ecological disorders of the intestinal flora in mice with HFD-induced obesity
[124]	Polysaccharides from *Ophiopogon japonicus*	Mice	Obesity, IR, T2DM	Improvement in IR in diabetic mice; decrease in *E. coli* and *Streptococcus intestinalis* populations; increase in *Bifidobacterium* populations
[125]	San huang shu ai soup	Mice	Ulcerative colitis	Improvement in probiotic *Lactobacillus* populations; regulation of the abundance of intestinal flora
[126]	Pingwei powder	Mice	Ulcerative colitis	Reduction in serum LPS, IL-17A, and IFN-γ mRNA levels; improvement of intestinal microbial abundance
[127]	Green tea extract	Mice	Obesity, IR	Downregulation of adipogenesis and inflammatory gene expression in the white adipose tissue of high-fat diet mice; restoration of changes in intestinal flora composition that are closely related to obesity and IR
[128]	*Portulaca oleracea*	Mice	Ulcerative colitis	Promotion of *Bifidobacterium* growth; reduction in excessive immune response in the intestine; regulation of intestinal micro-ecological disorders
[129]	FMT	Patients with intractable functional constipation	Refractory functional constipation	Improve the clinical symptoms of patients with refractory functional dyspepsia
[130]	FMT	Patients with ulcerative colitis	Ulcerative colitis	Alleviation of dysbiosis by the re-establishment of a new intestinal microecology in the gut
[131]	FMT	Patients with refractory irritable bowel syndrome	Refractory irritable bowel syndrome	No significant adverse effects and no infectious diseases in both groups after 3 months; FMT was determined to be clinically effective, safe, and reliable for the treatment of refractory irritable bowel syndrome.
[132]	FMT	Patients with depression	Depression	Potential improvement of depression that warrants more experimental studies
[133]	FMT	Patients with diarrhea associated with CDI	Diarrhea associated with CDI	Significant improvement in the cure rate of CDI-associated diarrhea, with multiple infusions having fewer adverse effects
[41]	*Lactobacillus plantarum* PS128	Athletes	Triathlon	Improvement in post-race anaerobic capacity and aerobic endurance; reduction in fatigue; alleviation of inflammation and oxidative stress
[134]	*Lactobacillus plantarum* TWK10	Healthy males	Aerobic endurance exercise	Significant reduction in serum lactate and ammonia levels after exhaustive exercise
[135]	*Bifidobacterium longum* OLP-01	Mice	Exercise performance	Improvement in grip strength and endurance of the forelimbs; significant reduction in the serum lactate, ammonia, and creatine kinase levels after acute exercise
[136]	*Lactobacillus salivarius* SA-03			
[137]	Atypical *Veillonella*	Mice	Endurance performance	Significant improvement in the endurance performance of the mice; 13% increase in exercise to exhaustion time compared to the control group
[84]	*Lactobacillus casei* IMC502	Bicyclists	Fatigue elimination	Reduction in serum creatine kinase levels after strenuous exercise after 4-week treatment; promotion of fatigue elimination; improvement in test performance
	*Lactobacillus rhamnosus* IMC501			
[138]	*Lactobacillus helveticus* L10	Endurance athletes	Antioxidant capacity of the body	Significant decrease in serum malondialdehyde levels in the probiotic group at the end of training when *Lactobacillus helveticus* L10 was administered daily over a 3-month training routine

HFD = high-fat diet; IR = insulin resistance; T2DM = type 2 diabetes mellitus; FMT = fecal microbiota transplantation; CDI = *Clostridium difficile* infection.

## Data Availability

Data are contained within the article.
